# Meeting Current and Future Aged Care Needs of Regional and Rural Communities Through the Multipurpose Service Model ‐ A Scoping Review

**DOI:** 10.1111/ajag.70217

**Published:** 2026-08-06

**Authors:** Linda Deravin, Melissa Hanson, Sarah Stenson, Catelyn Richards, Judith Anderson

**Affiliations:** ^1^ School of Nursing and Midwifery University of Southern Queensland Ipswich Queensland Australia; ^2^ School of Nursing, Paramedicine and Healthcare Sciences Charles Sturt University Bathurst New South Wales Australia

**Keywords:** ageing, nursing models, rural health services

## Abstract

**Objective:**

Despite more than two decades of implementation, there is limited peer‐reviewed evidence evaluating whether the multipurpose service (MPS) model effectively meets the contemporary health and aged care needs of rural and regional communities. This gap is particularly significant given recent government inquiries highlighting persistent inequities in rural health service access. The objective of this scoping review was to examine whether the key recommendations of the Royal Commission into Aged Care Quality and Safety and the New South Wales Parliamentary inquiry into Health outcomes for rural, regional and remote New South Wales support whether the multipurpose service model (MPS) is effective as a model of care for service delivery.

**Methods:**

A scoping review of five databases was undertaken. The first stage included title and abstract review followed by full text reviews. A thematic analysis was undertaken, informed by Braun and Clarke.

**Results:**

Nine articles met the criteria for review. Themes that were derived from the data that aligned with the two independent government reports were: MPS model and its infrastructure; funding; staffing and skill mix; and community engagement and needs.

**Conclusions:**

Overall, this scoping review established that the MPS model of care is an appropriate model to meet the recommendations of the two government reports. However, there needs to be further investment in staff development to extend scopes of practice when working in rural, regional and remote New South Wales.

## Policy Impact

Multi‐purpose services have been in place for over 20 years, yet no evaluation of this model has been undertaken. This scoping review affirms that this model is suitable for regional and rural communities in delivering health and aged care services to geographically isolated communities. However, more investment in staff professional development is required to support the delivery of service.

## 1 Introduction

Disparities in access to healthcare services and poorer health outcomes are long‐standing issues for rural and regional communities. Recent national reporting reinforces the persistence and scale of these disparities. The Australian Institute of Health and Welfare [1] identified that approximately 7 million people, around 27% of the Australian population, live in rural, regional and remote areas, where inadequate access to healthcare services remains a long‐standing systemic issue. The report highlighted a clear gradient of worsening health outcomes with increasing remoteness, with rural and remote populations experiencing higher rates of chronic disease, injury and mortality, as well as lower utilisation of primary healthcare services compared with metropolitan residents. These entrenched inequities underscore the need for service models that can sustainably deliver accessible, integrated care in geographically dispersed communities. Despite initiatives to increase access to services that support improving health outcomes, disparities continue [2].

A multipurpose service (MPS) is a distinct model of health and aged care service delivery that brings together a variety of services under one management structure. This integrated approach enhances continuity of care and ensures more efficient use of limited resources [3, 4]. The MPS program was initiated over 25 years ago to address these issues by combining federal and state health funding to provide a health service that would meet the needs of rural communities, particularly where standalone aged care services were not viable [4, 5]. In Australia today (latest data—2025), there are 183 MPS in existence; 66 of these are located in regional or rural New South Wales [6]. It is timely to review the efficacy of this service model in meeting current and future health demands of regional and rural communities.

Recently there have been two significant inquiries into healthcare systems within Australia that have focussed on people who live in regional and rural areas. Both inquiries were initiated in response to persistent and systemic inequities in rural health and aged care. Chronic workforce shortages, declining health outcomes with increasing remoteness and the ‘tyranny of distance’, which limits timely access to primary, acute and specialist services, have long affected rural communities. National data highlight higher rates of chronic disease, injury and mortality among rural residents, alongside reduced service utilisation. These entrenched challenges prompted governments to examine whether existing models, including the MPS model, were capable of delivering safe, sustainable and equitable care. In both inquiries, MPS were identified as a potential mechanism to address these challenges. The first of these two inquiries was the Royal Commission into Aged Care Quality and Safety [7]. In this report, the flexibility of the MPS model was recognised to address aged care service provision in communities. This model was seen as useful where standalone independent aged care providers might find the delivery of an aged care service a fiscal deterrent, or where aged care services were not being met even if there were existing aged care services within that local area. When aged care services are combined with acute care facilities, the opportunity to share the health workforce and other resources makes the MPS model an attractive economic option. This report also suggested that the MPS program should be expanded, and that governments should consider establishing MPS in areas where no acute care services exist [7]. This would require additional funding from governments to establish new facilities while also ensuring that existing MPS infrastructures are improved and updated to meet the current needs of local communities.

The second inquiry, the Parliamentary Inquiry into Health Outcomes and Access to Health and Hospital Services in Rural, Regional and Remote New South Wales [8], identified the need for the MPS program to continue. However, the report made specific reference to areas where this model was not meeting the needs of local communities. In this report, areas of concern were raised, including a lack of adequate staff to cover emergencies, under‐resourcing of medical staff in comparison with metropolitan counterparts, duplication and gaps in service delivery as a result of the federal and state health funding models, and a critical shortage of health professionals [8].

These government reports identified that regional and rural healthcare services require an appropriate service model to address the needs of local communities. The service model needs to be adequately resourced, funded and staffed to ensure its ongoing sustainability to meet future and current needs, and to address and improve regional and rural population health outcomes. The purpose of this scoping review was to establish whether the recommendations provided from the Royal Commission into Aged Care Quality and Safety and the Parliamentary Inquiry into Health Outcomes and Access to Health and Hospital Services in Rural, Regional and Remote New South Wales supported the need for a MPS model for regional and rural communities as a combined delivery of aged and acute care services.

## 2 Methods

As described by Munn et al. [9] a scoping review was selected as the preferred method for this review. The objective was to establish whether the findings within the literature supported the key aspects of the two independent government reports. These aspects included current infrastructure and if the model was fit for purpose; contributing factors to the funding gaps or in the provision of the delivery of care; the impacts of staffing models and if they were fit for purpose; and if the model was meeting the needs of the communities in which they were located.

### 2.1 Key Themes and Search Strategy

By reviewing sourced documents within the scoping review, the authors sought to gain an understanding WHETHER the recommendations and claims could be substantiated within the literature. Studies included in the review included concepts and Boolean terms as identified in Table 1.

**TABLE 1:** Search concepts and terms used in initial search.

Concept	Key words
Multi‐purpose services	“multipurpose service*” OR “multi‐purpose service*” OR “multi‐purpose health service*” OR “multi purpose service*” OR “MPS” OR “MPHS” AND
Rural and regional health services	“Rural” OR “Regional” OR “Remote” OR “Isolated” AND
Aged care	Aged care AND
Australia	“Australia”

The authors agreed on the inclusion and exclusion criteria (Table 2) and then conducted a review of each document sourced. Databases included in this search were CINAHL, MEDLINE(OVID), EMCARE, SCOPUS and INFORMIT (HEALTH COLLECTION). Although grey literature can broaden the scope of evidence in scoping reviews, the decision to exclude it was pragmatic and methodological. The review aimed to assess whether peer‐reviewed empirical literature substantiated the recommendations of the two government inquiries; therefore, a consistent standard of methodological rigour and reporting was required. Grey literature in the MPS context is highly heterogeneous, often descriptive, and frequently lacks sufficient methodological detail to allow meaningful comparison across sources. To maintain analytic consistency and ensure that included evidence met minimum reporting standards, only peer‐reviewed publications were included. As the first step, titles and abstracts were reviewed for relevance against the inclusion and exclusion criteria, with duplicates being removed. The next stage was a full article review by two members of the research team.

**TABLE 2:** Inclusion and exclusion criteria for the review.

Inclusion criteria	Exclusion criteria
English language	Not in English
Within 10 years—2014–2024	Outside specified time frame
Must include multi‐purpose services	Does not include multi‐purpose services
Australia	Outside of Australia
Government reports, including position papers	Fact sheets
Peer‐reviewed conference papers	Web pages
Peer‐reviewed articles	Conference abstracts
	Book chapters
	Editorials and opinion pieces

### 2.2 Results From Literature Search

**FIGURE 1:**
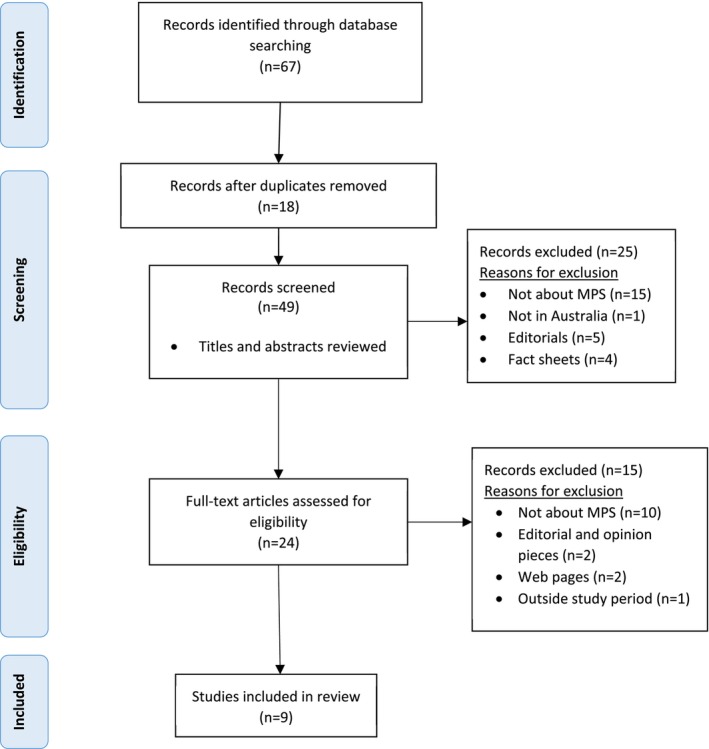
PRISMA diagram: Flowchart for inclusion of documents as per search criteria.

The initial database search yielded 67 documents. Duplicates (*n* = 18) were removed and then titles and abstracts of documents (*n* = 49) were independently reviewed by two members of the research team. When a conflict arose a third member of the team would provide resolution. After reviewing the titles and abstracts, a further 25 articles were removed as they did not fit within the inclusion criteria. This left a total of 24 papers for full text screening. The research team then completed a review of the documents and removed a further 15 papers, which left nine documents for inclusion in the scoping review. Figure 1 (PRISMA diagram) shows the flowchart of the document search [10].

### 2.3 Data Analysis and Theming

A six phase thematic analysis, informed by Braun and Clarke, was undertaken by the authors. This included (1) familiarisation with the data, (2) generating initial codes, (3) searching for themes, (4) reviewing themes, (5) defining and naming themes, and finally (6) producing the report [11]. The final nine articles were again reviewed by at least two authors within the team. Where there was conflict, the whole team came together to review and discuss. During this part of the review, all authors were asked to identify any themes that would align with the key concepts drawn out of the two independent government reports. The key concepts from the two government reports were: infrastructure and whether the model was fit for purpose, funding gaps or availability of resources to support the model, impacts of staffing in rural areas and if the needs of local communities were being met. Four main themes emerged from the review. These were an overall description of the MPS model and its infrastructure; funding; staffing and skill mix; and community engagement and needs (Table 3). These themes were interlinked and are described in more detail below.

**TABLE 3:** Study characteristics.

Author, year of publication	Title of article	Aim and methodology	Results, relevant findings	Themes
Anderson and Malone, (2014)	Suitability of the multipurpose service model for rural and remote communities of Australia	Aim: Overview of issues and significant aspects of the MPS model Methodology: Literature review of 38 relevant articles with common themes extracted and major themes identified	MPS model is responsive to community needs Provides flexible services Struggle for financial viability despite sharing staff across services and some economies of scale when combining acute and aged care Multiskilled staff required to meet broader needs of MPS Minimal staffing	Service model Funding Staffing Infrastructure
Bywood and Erny‐Albrecht, (2016)	Regionalisation of health services: Benefits and impact. Primary Health Care Research and Information Service Policy Issue Review	Aim: Evaluate regionalisation of health services Methodology: Rapid review	Although not regionalisation, MPS were seen to be beneficial Describes MPS model, economies of scale Conflicts between acute and aged care service provision Citizen engagement—steering group with citizen involvement Barriers to MPS implementation include accessing ongoing funding, recruitment and retention of staff, and burdensome administrative processes	Service model Funding Staffing Infrastructure
Gilbert, Owusu‐Addo, Feldman, Mackell, Garratt, Brinjnath, (2020)	Models of integrated care, health and housing	Aim: Report prepared for the Royal Commission into Aged Care regarding integrated models of care for older people in Australia Methodology: Interviews with key care providers	Models of service delivery should reflect the different needs of different communities Pooling of funds Purpose is to address gaps in acute care and aged care services in rural and remote areas Governance structures should include community representatives MPS provide employment opportunities for towns Sometimes acute and medical care is prioritised over psychosocial needs of residents	Service model Funding Staffing Infrastructure Community involvement
Henderson, Willis, Xiao, Tpffoli, Verrall, (2016)	Nurses' perceptions of the impact of the aged care reform on services for residents in multi‐purpose services and residential aged care facilities in rural Australia	Aim: To understand the impact of aged care reform on nursing staff that work within MPS and rural aged care facilities Methodology: Purposively sampled participants undertaking semi‐structured interviews	Funding and resource shortfalls impact on the delivery of care Workforce issues were raised such as inadequate staffing levels, skill mix and knowledge gaps in current staffing Resource shortages related to funding compromised the quality of care offered to residents Increased acuity in patients not matched by skill and level of experience of staff	Funding Staffing
Malone and Anderson, (2015)	Understanding the need for the introduction of the multipurpose service model in rural Australia	Aim: To gain an understanding of the reason for implementing the MPS model in rural and regional areas Methodology: Literature review	Little evidence on effectiveness of service model Activity‐based funding not suitable for MPS Service fragmentation caused by funding divided by State and Federal Governments Needs to meet and adapt to community needs and requires community and staff consultation Collaboration between levels of government is needed to support the funding model and development of MPS facilities Communities need to be involved in the development of MPS	Service model Staffing
O'Callaghan, (2017)	Extending the multipurpose service concept to improve children's outcomes	Aim: To trial an expanded MPS conceptual model to provide better care to children and not just aged care Methodology: Case study including observation, unstructured interviews and meetings	Need for flexible funding that is based on the needs of the community/population and not on activity‐based funding Service should go beyond aged and acute care MPS provides a good model for aged care service delivery Need for skilled and collaborative workforce	Service model Funding model
Savy, Warbuton and Hodgkin, (2017)	Challenges to the provision of community aged care services across rural Australia: Perceptions of service managers	Aim: To ascertain the perception of rural aged care service managers in hiring a diverse workforce Methodology: Qualitative study using 11 semi‐structured interviews	Community aged care sector is a complex place. Diversity in the skill of workers The need for flexible, generalist workers in rural services that offered multiple care types Attracting and retaining staff Secure wages, support and pathways for promotion may help to address retention	Staffing workforce issues Although this study was not exclusively related to MPS, it did include managers of MPS
Sheehan, (2016)	Position paper: Reshaping the multipurpose service (MPS) model in NSW	Aim: Guide future planning of MPS in NSW Methodology: Discussion paper	MPS have had a positive impact Predicted growth in older Australians living in rural areas increases need for MPS Recommends an integrated model of care for sustainability Change in aged care level distinction could lead to a higher demand for MPS aged care places	Service model; Funding model
Victoria Auditor‐General's Office, (2018)	Community health program: Independent assurance report to Parliament	Aim: Audit of community health programs in Victoria, including 1 MPS (Orbost) Methodology: Audit, including interviews, reviews of strategies, site visits and analysis of Community Health Minimum Dataset by the Victorian Auditor‐General's Office (VAGO)	Funding of services is based on historical data funding model is potentially outdated—2007 Access to services Need to collect purposeful data to support funding and infrastructure Difficulty attracting health specialists Difficulty with transport	Workforce issues Funding

## 3 Results

### 3.1 The MPS Model and Infrastructure

The MPS model is an integrated care model designed to address the health needs of small rural and remote communities. Within the model, acute care, primary health care, aged care and other healthcare services are integrated as appropriate to the local community. The MPS model allows flexibility in what services it provides in meeting community needs, and by improving community access to a range of services [3, 4, 12, 13, 14]. The model has been particularly well adapted to aged care services. However, the model could also be effectively extended to meet the needs of younger people within the community [15]. Although the model frequently combines aged care and acute services, the dual funding source can lead to conflict regarding staff mix and issues related to the multidisciplinary skills required for this to be effective [3, 4, 12, 13, 16].

Multi‐purpose services attempt to involve the community in decision‐making [3, 4, 12] but differences in community perception and actual use of services can lead to conflict. Victorian MPSs are governed by independent boards, increasing their independence and enhancing the role of the community within their management, which seems to make them more effective in understanding and addressing community needs than those in other states [12, 13]. The development of an MPS is often limited by capital funding, resulting in the need to adapt existing buildings or delivery of services in more than one site and creating issues with economies of scale and staffing, which can undermine the implementation of the model [4, 13].

### 3.2 Funding

In an MPS, activity‐based funding is used to allocate resources for the acute care component of service delivery, and an average funding level is allocated to the aged care component of service delivery [16]. This does not allow care providers in the MPS to account for differing acuity and dependency levels of aged care residents. Prior to 2022, residential aged care facilities (RACF) utilised the aged care funding instrument (ACFI), which provided an incentive for aged care providers to accept people into aged care who have higher dependency levels [16]. This resulted in MPS facilities struggling to remain financially viable.

As funding is pooled from both the federal and state government, it has been shown that even with the best intent, there continues to be service delivery gaps, and in some communities a duplication of services. O'Callaghan [15] identified that even though the MPS model has allowed significant improvements in the delivery of primary care services and a coordinated approach to health and aged care services, gaps still exist in primary care service delivery, and highlighted the potential to increase services in areas such as child and family care. Sheehan [17] agreed that an expansion of the MPS model and the services that it can provide should be considered to ensure future sustainability. The model of an MPS should also be responsive to the needs of the local community rather than the implementation of a standardised model [4, 13].

As some MPS facilities are now over 20 years old, and community needs change, there also needs to be capital investment in updating and improving existing infrastructure, such as dementia‐specific units [16]. When new MPS facilities are earmarked for implementation, careful consideration must be given to avoid duplication of existing aged care or primary healthcare services. Duplication of services only contributes to fragmentation of care and the dilution of resources. Communities, key stakeholders and governments should work together to develop a service model that meets the community's needs both now and into the future [3, 5].

### 3.3 Staffing and Skill Mix

Multi‐purpose services are designed to combine a flexible range of services focussed on the unique needs of rural and remote communities [4, 12, 13]. As such, staff in MPS are required to have wide‐ranging experience and be competent in an extensive range of skills, described as a *generalist* role. This role enables communities with MPS to be provided with much‐needed flexibility in the provision of health care through sharing of staff across the boundaries of care provision, especially in isolated rural communities where it is difficult to recruit and retain specialists [4, 13, 14, 18]. The lack of specialists to fulfil an MPS's services results in patients often having to travel to neighbouring services, which provides issues of its own due to the transport issues of isolated rural and remote communities [14]. As a result of difficulties recruiting and retaining experienced staff, many MPS rely on graduates to fulfil staffing needs, although most only stay and work in the community for a short period before leaving [14, 16]. Savy et al. [18] found that this was in contrast to the MPS managers' preference to employ older workers who have previous employment and life experience, as well as a perceived maturity and adaptability within the workplace. The current difficulties described in recruiting and retaining experienced staff for MPS will only deepen in the future, with issues related to attrition of the current ageing workforce, an ageing and increasingly frail population requiring care—especially in rural areas [3], and the negative image of working in aged care [12, 16, 18].

Conversely, the *generalist* role of MPS nurses was found to have detrimental effects on aged care residents, where nurses tended to focus on medical aspects of care to the detriment of social care [16]. Henderson et al. [16] found that higher registered nurse ratios, as opposed to dedicated aged care nurses, were linked to poor continence outcomes and declines in activities of daily living. This supports the need for MPSs to employ care staff with dedicated specialty knowledge and education, especially in areas such as dementia, palliative and gerontological care.

As mentioned previously, funding issues were also associated with the staffing shortfalls in MPS [16]. Multi‐purpose services often have minimum staffing of two nurses per shift [4]. This means that care can be difficult to manage with multiple areas requiring attention. However, aged and acute care seem to be areas that are neglected most often as emergency department presentations require multiple staff members to attend, drawing these nurses away from their allocated clients [4, 16]. This situation brings fresh concerns with aged care staff feeling apprehensive about having to provide care for acute and emergency patients as a result of a lack of experience and skills in those areas [3]. Staff shortages often meant there was an inability to release staff for ongoing professional development and leave, resulting in educational deficits within MPS staff [5, 16].

### 3.4 Community Engagement and Needs

The MPS model aims to address the individualised needs of the rural and remote communities they serve. A ‘one‐size‐fits‐all’ approach is not effective in meeting individual communities' needs [3]. The importance of community consultation cannot be understated. Multi‐purpose services, staff and delivery models must be tailored to the needs of the community [3, 4, 13, 16, 18], and MPS must provide a sustainable model that meets the needs of rural communities when community partnerships are fostered and encouraged, and the service is responsive to the evolving health of the community [13, 14, 16, 18].

The MPS model often includes residential aged care and acute services [4, 13, 14], but there is the opportunity to expand services to include multidisciplinary health professionals that can address a wide range of needs for the varying demographic groups within the community. It is essential that services and funding are not simply based on historical data, as communities evolve and change over time. Community consultation must therefore be ongoing, such as having community representation on the governing board [14]. Models that are directly driven by the needs of the community and are able to adapt to the evolving needs of the community are most effective [4, 13, 14].

## 4 Discussion

This scoping review identified four interrelated themes that collectively describe how the MPS model functions within rural and remote communities: (1) the structure and implementation of the MPS service model and its infrastructure; (2) funding arrangements and their implications for service delivery; (3) staffing and skill mix challenges; and (4) community engagement and responsiveness to local needs. These themes provide the framework for the following discussion, which examines how the existing literature aligns with the recommendations of the Royal Commission into Aged Care Quality and Safety and the Parliamentary Inquiry into Health Outcomes and Access to Health and Hospital Services in Rural, Regional and Remote New South Wales.

### 4.1 The MPS Model and Infrastructure

The MPS program began in 1993 to address rural health problems [19]. This scoping review identified that little has been published about the model and how it functions despite more than 20 years passing since it began. Although MPSs have a variety of aims, the ability to pool funding from a variety of sources, economies of scale and shared multiskilled staff are important features related to their success, particularly when they are supported by an initial funding boost to build (or renovate) suitable facilities. This integrated care model is most suitable for small towns (< 5000 inhabitants) that are unable to financially support individual health services, often making this model their only option for sustainable local health services. For this reason, amalgamating State and Federal funding in the form of acute care and aged care frequently has positive results [4, 12, 17, 19, 20].

### 4.2 Funding and Sustainability

As noted in both inquiries, the funding model that was in place to support the MPS program required further review [5, 7]. A new aged care funding model was introduced in 2022 to try to address this requirement through fixed, variable and one‐off payment subsidies. Despite these financial benefits, there are some drawbacks of the model such as flexibility in meeting community needs and involving community in decision‐making. Although MPSs differ in the services offered, this does not necessarily mean that there is ‘flexibility’ of services that are provided. Sometimes it more accurately reflects which health practitioners incidentally live within the community. This can lead to a continuation of gaps in services which are prominent in primary health care, services for younger people [15] and First Nations Peoples [7]. In contrast to the international acceptance that models of integrated care which centre on primary health care are efficient and effective [12], the MPS program seems to focus on aged care.

Even with the benefits of pooled funding, issues arise. Aged care services within a MPS receive an average funding level rather than being able to claim for differing acuity and dependency as independent aged care services are able to do, which can be an incentive to accept people with a low level of dependence rather than those with higher needs [7, 16, 19]. The MPS model does have success, leading to some calls for expansion of the model and its services [7, 17], but also calls for further investment to ensure that those MPS which are already in existence have an opportunity to change with the changing needs of their communities and update existing infrastructure that is now ageing [7, 16]. Woods et al. [19] indicated that there have been limited applications to develop MPS from Australian States. The Royal Commission into Aged Care Quality and Safety Final Report [5] found that there is a continuing decline in working‐age people, combined with an increasingly ageing population. These conditions will worsen by 2058, raising concerns in relation to the demand that will be put on already stretched rural and remote services. The ‘Health outcomes and access to health and hospital services in rural, regional and remote New South Wales’ Parliamentary Inquiry [8] similarly found that rural, regional and remote NSW was experiencing challenging and complex issues surrounding healthcare delivery that were significantly linked to workforce challenges such as staff shortages [8].

### 4.3 Staffing, Skill Mix and Workforce Pressures

This review found that the MPS model is not immune to the continuing struggle with staffing shortfalls [3, 4, 5] that threaten the viability of the MPS model. Staffing issues are further hampered by MPS models being seemingly focussed on aged care, with concerns related to the negative image of working in aged care impacting recruitment [12, 14, 16, 18].

### 4.4 Community Engagement and Local Responsiveness

The MPS model provides local employment opportunities for people in rural and remote communities [12]. The flexible care model of MPSs means that most staff are required to be ‘rural generalists’, having skills in multiple areas such as emergency, acute care and aged care. This provides employees of these facilities with rich and potentially rewarding careers that they likely would not encounter in metropolitan areas.

### 4.5 Limitations

There is a paucity of evidence regarding multi‐purpose services in Australia which limited the interpretation of the validity of this model. The exclusion of grey literature, despite its relevance to rural health policy and service models, may have limited the breadth of contextual and operational insights available, particularly given the small evidence base on MPS. Future reviews incorporating grey literature may provide a more comprehensive understanding of implementation and policy considerations.

## 5 Conclusions

The Royal Commission into Aged Care Quality and Safety [7] and the Parliamentary Inquiry into Health Outcomes and Access to Health and Hospital Services in Rural, Regional and Remote New South Wales [8] identified that regional and rural healthcare services require an appropriate service model to address the needs of local communities. The literature included in this review indicates that the MPS model is appropriate to meet those requirements in small rural and remote communities. It identifies the need for ongoing funding to ensure that existing and new infrastructure and service demands are being met and that there is continuing government support for this model of care. This review highlighted that the MPS model with its flexible multiskilled staff makes good use of limited staff in rural areas yet could also benefit from further investment to develop the broad range of skills required by these staff members. Community engagement is also supported by the MPS model, with the Victorian implementation of the model providing greater engagement with the community and thereby further flexibility to meet community needs. This is a concept that other states could embrace to enhance their ability to meet community needs.

## Funding

The authors have nothing to report.

## Ethics Statement

The authors have nothing to report.

## Conflicts of Interest

The authors declare no conflicts of interest.

## Data Availability

The authors have nothing to report.
